# Stroke and physiological relationships during the incremental front crawl test: outcomes for planning and pacing aerobic training

**DOI:** 10.3389/fphys.2023.1241948

**Published:** 2023-08-14

**Authors:** Tiago A. F. Almeida, Mário C. Espada, Danilo A. Massini, Anderson G. Macedo, Eliane A. Castro, Cátia C. Ferreira, Joana F. Reis, Dalton M. Pessôa Filho

**Affiliations:** ^1^ Graduate Programme in Human Development and Technology, São Paulo State University (UNESP), Rio Claro, Brazil; ^2^ Department of Physical Education, São Paulo State University (UNESP), Bauru, Brazil; ^3^ CIPER, Faculdade de Motricidade Humana, Universidade de Lisboa, Lisboa, Portugal; ^4^ Instituto Politécnico de Setúbal, Escola Superior de Educação, Setúbal, Portugal; ^5^ Life Quality Research Centre (LQRC—CIEQV, Leiria), Complexo Andaluz, Rio Maior, Portugal; ^6^ Research Group in Optimization of Training and Sport Performance (GOERD), Faculty of Sports Sciences, University of Extremadura, Cáceres, Spain; ^7^ Faculdade de Motricidade Humana, Universidade de Lisboa, Lisboa, Portugal

**Keywords:** stroke mechanics, oxygen uptake, aerobic conditioning, exercise zones, swimming

## Abstract

**Purpose:** This study aimed to evaluate the physiological responses associated with the stroke length (SL) and stroke rate (SR) changes as swimming velocity increases during an incremental step-test. Moreover, this study also aimed to verify if SL and SR relationships toward maximal oxygen uptake (V̇O_2max_), gas respiratory compensation point (RCP), exchange threshold (GET), and swimming cost can be applied to the management of endurance training and control aerobic pace.

**Methods:** A total of 19 swimmers performed the incremental test until volitional exhaustion, with each stage being designed by percentages of the 400 m (%v400) maximal front crawl velocity. V̇O_2max_, GET, RCP, and the respective swimming velocities (v) were examined. Also, the stroke parameters, SL, SR, the corresponding slopes (SLslope and SRslope), and the crossing point (Cp) between them were determined.

**Results:** GET and RCP corresponded to 70.6% and 82.4% of V̇O_2max_ (4185.3 ± 686.1 mL min^-1^), and V̇O_2_ at Cp, SLslope, and SRslope were observed at 129.7%, 75.3%, and 61.7% of V̇O_2max,_ respectively. The swimming cost from the expected V̇O_2_ at vSLslope (0.85 ± 0.18 kJ m^-1^), vSRslope (0.77 ± 0.17 kJ m^-1^), and vCp (1.09 ± 0.19 kJ m^-1^) showed correlations with GET (r = 0.73, 0.57, and 0.59, respectively), but only the cost at vSLslope and vCp correlated to RCP (0.62 and 0.69) and V̇O_2max_ (0.70 and 0.79).

**Conclusion:** SL and SR exhibited a distinctive pattern for the V̇O_2_ response as swimming velocity increased. Furthermore, the influence of SL on GET, RCP, and V̇O_2max_ suggests that SLslope serves as the metabolic reference of heavy exercise intensity, beyond which the stroke profile defines an exercise zone with high cost, which is recommended for an anaerobic threshold and aerobic power training. In turn, the observed difference between V̇O_2_ at SRslope and GET suggests that the range of velocities between SL and SR slopes ensures an economical pace, which might be recommended to develop long-term endurance. The results also highlighted that the swimming intensity paced at Cp would impose a high anaerobic demand, as it is located above the maximal aerobic velocity. Therefore, SLslope and SRslope are suitable indexes of submaximal to maximal aerobic paces, while Cp’s meaning still requires further evidence.

## 1 Introduction

Swimming is a sport that requires high technical proficiency, which, alongside the cost of swimming, is a factor that will decisively determine the training and competition performance (Wakayoshi et al., 1995; Silveira et al., 2019; Zacca et al., 2020). In training planning, it is important to incorporate the energetic balance that aligns with the specific race requirements when determining the duration of work intervals (Zamparo et al., 2005; Almeida et al., 2021), and swimming coaches should also assume that the time limit and maximal aerobic velocity (MAV) are influenced by stroking parameters (Wakayoshi et al., 1993; Almeida et al., 2022). In this sport, integrating precise measurements with less time-consuming or intrusive methods supports the ongoing exploration of applied exercise physiology in sports training (Espada et al., 2015; Zacca et al., 2019). This pursuit is driven by the recognition that performance improvement is intricately linked to the accurate identification of exercise intensity domains, thereby facilitating the optimization of daily training (Toussaint and Hollander, 1994; Nomura and Shimoyama, 2003; Espada et al., 2021).

Recently, Mezêncio et al. (2020) emphasized that in swimming, as a cyclic sport, the effectiveness of optimal limb coordination relies on its maintenance across consecutive stroke cycles. However, previous studies have demonstrated that inter-limb coordination in swimming is influenced by alterations of the stroke length (SL) and stroke rate (SR), with increasing swimming velocity and exhaustion (Alberty et al., 2005; Seifert et al., 2014). Indeed, if the upper limbs can contribute to approximately 85% of the propulsive force in swimming (Bartolomeu et al., 2018), the SL and SR relationship might be considered an index to analyze the coordination mode and the mechanical power output at a given velocity (Alberty et al., 2009; Ribeiro et al., 2017), thus also influencing energetic requirements (Barbosa et al., 2008). These were probably the reasons supporting the assumptions that swimmers dedicate much of their effort to increasing the propulsive force of the upper limbs (Crowley et al., 2017) and that SL and SR alterations with swimming velocity (v) progression were associated with aerobic pace indexes, such as physiological thresholds (Wakayoshi et al., 1993; Carvalho et al., 2020).

Despite the evidence, the question of whether SL and SR might be applied to plan and control aerobic training, as well as monitor the improvements in endurance ability, still requires further physiological characterization to determine which variable of exercise intensity should be parameterized to SL and SR and whether these stroke variables differ with regards to the influence on a given physiological response. For example, the peak of the SL response to increase in swimming velocity typically falls within the range of 65%–70% of the 200 m swimming performance, being better related to the lactate threshold (LT) than to the onset of blood lactate appearance (OBLA) (Nomura and Shimoyama, 2003). On the other hand, when SR increases above the critical values (e.g., above ∼50 strokes min^-1^), the spatial–temporal stroke coordination reaches the superposition mode (i.e., the propulsive phases of the two arms overlap) (Seifert et al., 2014). This adaptation not only increases propulsion but also requires an efficient technique to avoid the overdemand on the drag force, mechanical power, and energy output (Barbosa et al., 2008; Alberty et al., 2009; Ribeiro et al., 2017).

Indeed, SL and SR responses differed from moderate to heavy or severe swimming intensities with the downward and upward turn points, respectively, for SL and SR coinciding with the lactate turn point (i.e., lactate threshold) (Carvalho et al., 2020). Moreover, the decline in both propelling efficiency and swimming cost are also aligned with the reduced SL and increased SR values in swimming intensities reaching maximal aerobic and anaerobic paces (Barbosa et al., 2008; Ribeiro et al., 2017), which are circumstances related to ongoing fatigue development (Alberty et al., 2005). Consequently, analyzing SL and SR profiles can offer valuable insights into the physiological exercise domain, aiding in training management and assessing improvements in technical proficiency and contributing to achieving optimal performance.

However, studies relating to stroke, physiological parameters, and cost have postulated that swimming at maximum individual speed or under exhausting conditions requires a maximum frequency of strokes, imposing mechanical and physiological restrictions on the emergence of a stroke movement pattern that is operationally robust to accommodate the task requirements economically (Aujouannet et al., 2006; Alberty al., 2014). Therefore, competitive swimmers must associate the increase in the stroke rate with the improvement in propulsive efficiency to avoid velocity reduction since the cost (e.g., the ratio between oxygen costs and SR or velocity) relates to different indexes of maximal and submaximal aerobic paces (Wakayoshi et al., 1995; Ribeiro et al., 2017). On the contrary, SL should increase rather than SR to improve the swimming velocity with no additional demand on energy metabolism during the high-intensity swimming pace (Wakayoshi et al., 1993; Barbosa et al., 2008). Since studies have explored the relationships between stroke mechanics, energetics, and performance, the association of stroke mechanics toward the oxygen uptake (V̇O_2_) responses ranging from submaximal to maximal rates during swimming remains unstudied, despite its suitability for exercise intensity management.

The rationality of the current study is, therefore, to provide additional evidence on the association of SL and SR to the determinants of endurance performance (such as GET, RCP, V̇O_2max_, and swimming cost) and consequently offer feasible and reliable references for swimming training planning and pace strategies for different swimming races. Moreover, the association between stroke mechanics and exercise intensity indexes is expected to support the lack of physiological information about the role of SL and SR responses in managing swimming zones with tolerable and exhaustive metabolic profiles. Hence, during a maximal front-crawl progressive test, this study aims to verify how SL and SR profiles would relate to V̇O_2_ responses. Moreover, this study analyses the suitability of SL (SLslope), SR (SRslope), and the crossing point (Cp) to control the aerobic pace by locating these indexes into exercise zones and verifying the correlation level to the swimming velocity at GET, RCP, and V̇O_2max_. This study hypothesized that the SL and SR slopes (i.e., turn points) during an incremental exercise will demand different physiological responses, and when considering that the reduction of SL with the increasing exercise intensity is associated with the underwater faster hand velocity (Wakayoshi et al., 1995; Barbosa et al., 2008; Matthews et al., 2017), it will probably show more influence than SR on indexes of metabolically costly exercise intensities.

## 2 Materials and methods

### 2.1 Participants

In total, 19 well-trained male endurance swimmers (18.5 ± 5.6 years old, 66.3 ± 9.0 kg body weight, 176.1 ± 8.3 cm height, and 13.1% ± 4.0% body fat mass) with the best front crawl performances at the 50, 100, and 200 m representing 575 ± 95, 599 ± 100, and 588 ± 94 FINA points, respectively, participated in the study. All swimmers included in the study were actively participating in competitive training programs for a minimum of three consecutive annual seasons, with a mean swimming training volume of 25 km per week^-1^. The training was scheduled with 10.8% at very hard, 8.1% at hard, and 81.1% at light-to-moderate workload intensities. These intensity levels were determined based on exertion level zones and the thresholds for three blood lactate accumulation zones (Gonzalez-Rave et al., 2021).

Before participating in the research, all subjects and their respective guardians (when under 18 years old) provided informed consent by signing a consent form. The research conducted in this study was approved by the local University Ethical Committee in Human Research from São Paulo State University (UNESP-CAAE:02402512.7.0000.5398) in accordance with the principles outlined in the 1975 Declaration of Helsinki, ensuring the ethical considerations and standards for human research participants.

### 2.2 Study design

The swimmers performed an incremental step-test comprising five to eight 300 m stages with 30 s rest in between, until volitional exhaustion (i.e., stop before the stage end or unable to maintain the required pace) to evaluate V̇O_2max_, GET, and RCP. Participants were given instructions to refrain from engaging in intense training sessions and consuming beverages containing caffeine or alcohol for a minimum of 24 h prior to the experimental sessions.

### 2.3 Procedures

Based on the swimmers’ 400 m maximal front crawl velocity (v400 m), an incremental intermittent step-test was planned with seven stages of 300 m. The front crawl swimming velocity at the first stage was set at 70% of v400 m and progressed by 5% at each stage. The pace control during the steps was monitored by an experienced professional, who provided feedback to the swimmer every 50 m. The highest V̇O_2_ (averaged 9 s after a 3 s filter of the V̇O_2_ breath-by-breath data) achieved during the incremental step-test was considered to be V̇O_2max_. For V̇O_2max_ confirmation, two criteria were considered: 1) a V̇O_2_ plateau phenomenon (variation <150 mL min^-1^) despite increments on intensity; 2) a respiratory exchange ratio (RER) above 1.10 (Poole et al., 2008).

During all tests, pulmonary gas sampling was collected through a portable breath-by-breath apparatus (K4b^2^, COSMED, Rome, Italy), coupled to the swimmer by a specific respiratory snorkel and valve system (Keskinen et al., 2003). Tests were conducted in the front crawl technique, using open turns, in a 50 m indoor swimming pool. GET and RCP were visually determined by two independent researchers through the analysis of V_E_·VCO_2_^−1^, V_E_·V̇O_2_^−1^, PETCO_2_, and PETO_2_ parameters. GET determination was considered to increase in V_E_·V̇O_2_^−1^ and PETO_2_, without a concomitant change in V_E_·V̇CO_2_^−1^ and PETCO_2_, respectively, and RCP was identified before the continuous increase in V_E_·V̇O_2_^−1^ and V_E_·VCO_2_^−1^ with a concomitant reduction in PETCO_2_ (Beaver et al., 1986). vGET, vRCP, and vV̇O_2max_ were the corresponding velocities at the stage where each physiological response was observed.

SR, expressed in strokes per second (str·s^-1^), was calculated through the equation (SR = 60/stroke duration) (Craig and Pendergast, 1979), and SL, with the equation (SL = v/SR in m∙cycle^−1^). In order to adjust SL and SR (y) to v and V̇O_2_ (x) for each participant’s incremental step-test, a second-order polynomial function was applied. The determination of the slope (i.e., the turn point or the vertex of the function) after which the quadratic function starts to decrease (if a < 0, or the maximum SL) or increase (if a > 0, or the minimum SR) was assessed for the y (-D/4a, where D = b^2^ - 4ac), while the corresponding point for the x (-b/2a) assessed v and V̇O_2_ at each maximum SL or minimum SR (e.g., vSL_slope_, vSR_slope_, V̇O_2_SL_slope_, and V̇O_2_SR_slope_). v and V̇O_2_ at Cp (vCp and V̇O_2_Cp) were assessed by considering the intersection point between SL and SR adjustments to v and V̇O_2_. The best adjustments between V̇O_2_ (y) and v (x) were analyzed. The swimming cost was calculated at vSL_slope_, vSR_slope_, and vCp by applying the individual relationship between V̇O_2_ and v, considering the arbitrary caloric constant for V̇O_2_ (20.9 kJ), i.e., each 1 mlO_2_ is taken as equivalent to 20.9 J; therefore, each 1 W kg^-1^ is equivalent to 2.871 mlO_2_ kg^-1^ min^-1^ with the cost (in kJ m^-1^), considering the rate between kW and velocity (Massini et al., 2021).

### 2.4 Statistical analysis

The Shapiro–Wilk test tested the normality of data, which was expressed as mean ± standard deviation (M ± SD), and the variance described to the 95% confidence interval (95%CI). The mean values of V̇O_2_ and v corresponding to GET and RCP were contrasted to those of v and V̇O_2_, corresponding to SL_slope_, SR_slope_, and Cp using the ANOVA test (one-way, with LSD as *post hoc* analysis). The swimming cost at vSR_slope_, vSL_slope_, and vCp was correlated to vV̇O_2max_, vGET, and vRCP by Pearson`s coefficient, respectively, and values were interpreted as 0.90–1.00 [very strong], 0.70–0.89 [strong], 0.40–0.69 [moderate], 0.10–0.39 [weak], and 0.00–0.10 [negligible] (Schober et al., 2018). The post-test sample power was calculated using G*Power 3 software taking into account the results of Pearson’s coefficient, the actual sample size (N = 19), and the specified significance level (α = 0.05), following the methodology described by Faul et al. (2007). Data analysis was conducted using the Statistical Package for Social Sciences (SPSS 26.0, SPSS. Inc., Armonk, NY, United States).

## 3 Results

**FIGURE 1:**
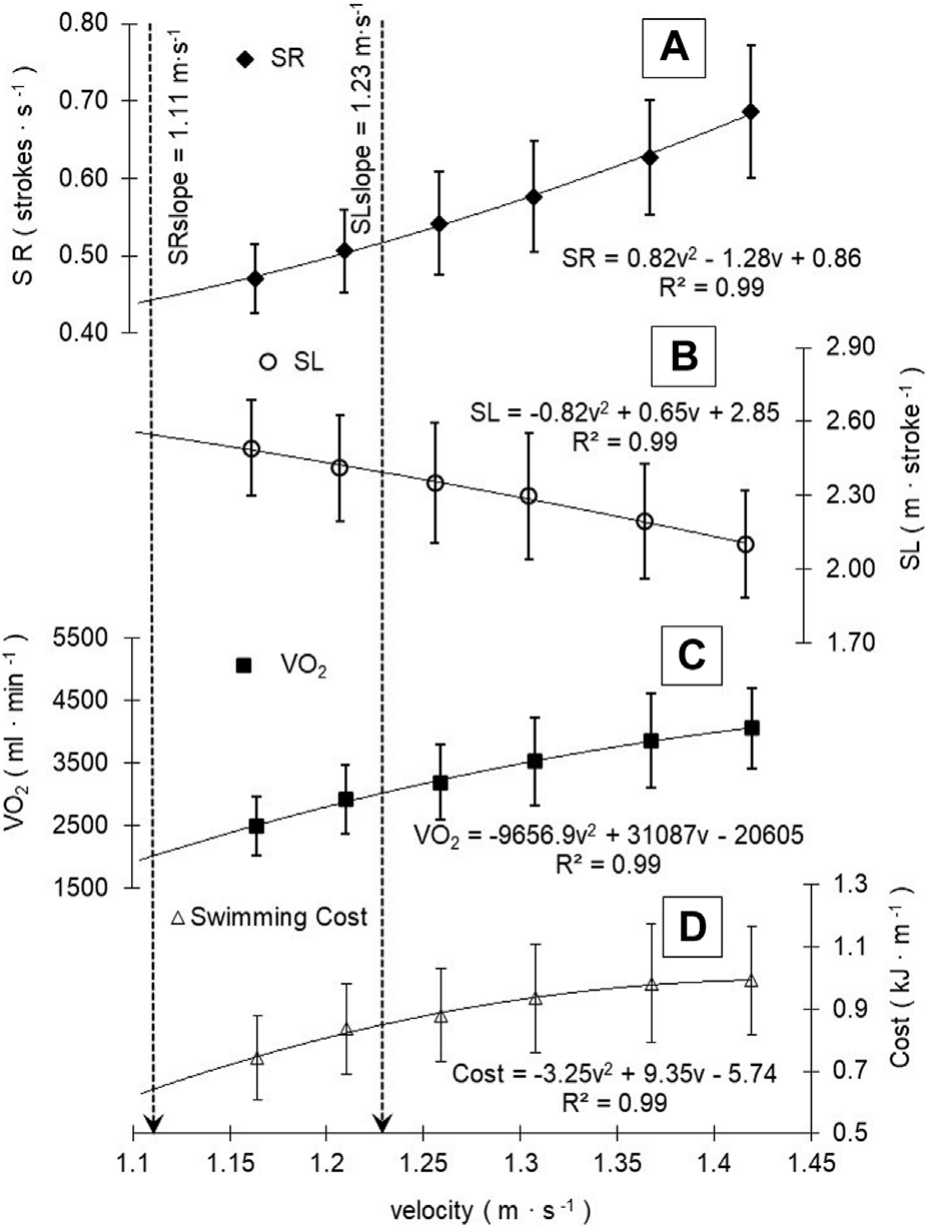
SR, SL, V̇O_2_, and swimming cost profiles (**A**, **B**, **C**, and **D**, respectively) during swimming velocity progression. Mean group and standard deviation values were plotted, and the best slope adjustments was depicted. Black dashed vertical lines are demarcating SL SR slopes during the increment of swimming velocity.

Figure 1 shows the adjustments in V̇O_2_ and stroke parameters as the swimming velocity progresses during the incremental step-test. The second-order polynomial relationships between SR and SL with v showed the higher coefficient of adjustments for each performance. Group adjustment reflects a trend for the increase of SR after 1.11 ± 0.18 m s^-1^ (95%CI: 1.03–1.19 m s^-1^) and the decrease of SL after 1.23 ± 0.13 m s^-1^ (95%CI: 1.17–1.29 m s^-1^), matching each other (Cp) at 1.66 ± 0.14 m s^-1^ (95%CI: 1.17–1.29 m s^-1^). Figure 1 shows the profiles across the swimming velocity progression of SR, SL, V̇O_2_, and swimming cost during an incremental step-test.

**FIGURE 2:**
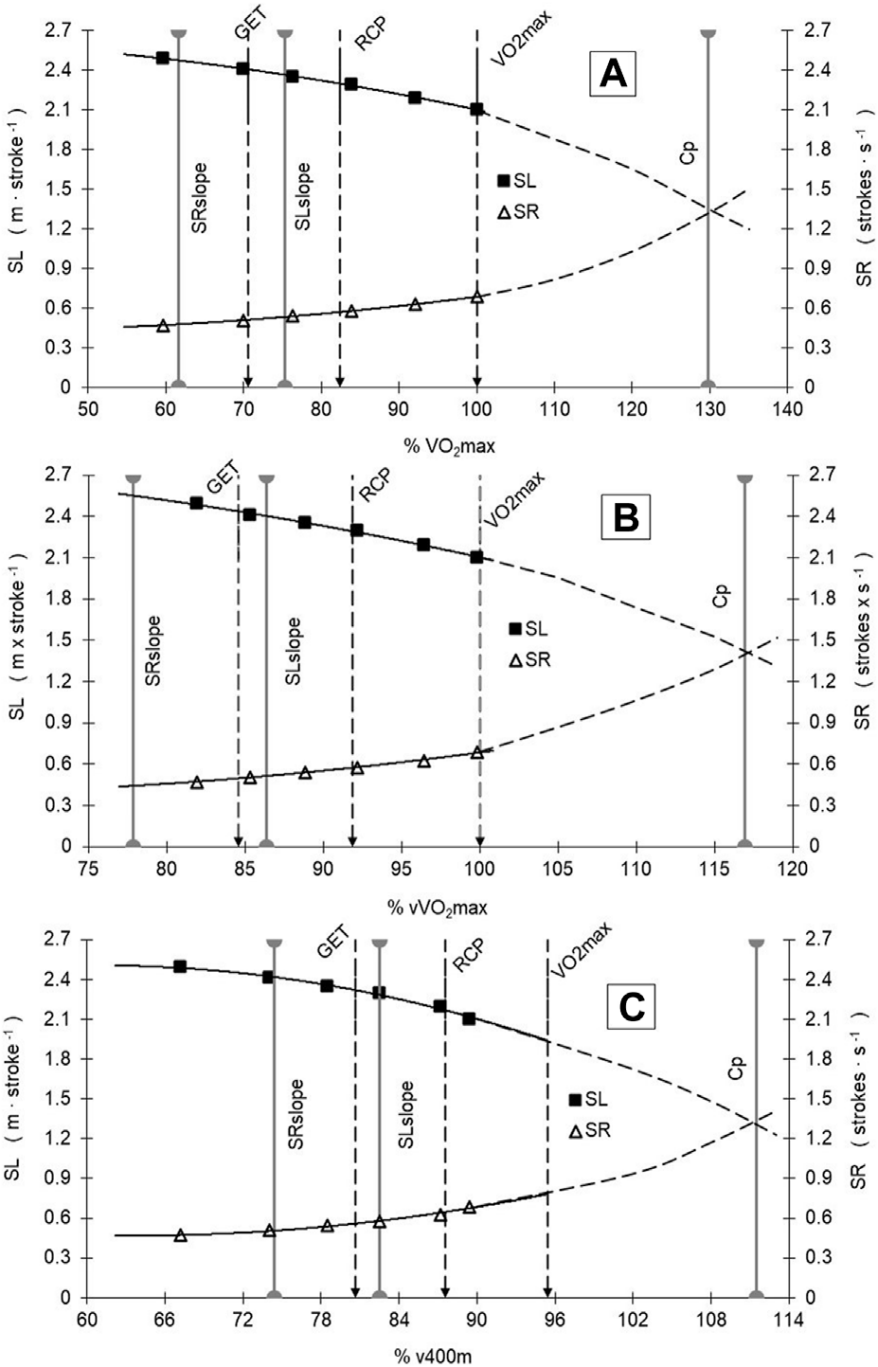
SR and SL vs. V̇O_2_ [in %V̇O_2max_ and %vV̇O_2max_—Panels **(A,B)**] and %v400 m Panel **(C)** responses during the incremental test and swimming performance in the 400 m race. See text for further details. Gray solid vertical lines are demarcating SL and SR slopes and the Cp point.

When related to the mean V̇O_2_ response to v increments, SL tends to decrease after 3141.9 ± 853.8 mL min^-1^ (95%CI: 2758.0–3525.8 mL min^-1^), SR increased after 2532.5 ± 674.0 mL min^-1^ (95%CI: 2229.4–2835.6 mL min^-1^), and project V̇O_2_ at Cp was 5415 ± 1208.2 mL min^-1^ (95%CI: 4871.7–5958.2 mL min^-1^). The peak of SL, after which it tends to decrease (2.34 ± 0.32 m∙cycle^−1^, 95%CI: 2.20–2.49 m∙cycle^−1^), occurred at different exercise domains and race paces, where SR begins to increase (0.51 ± 0.13 str·s^-1^, 95%CI: 0.45–0.56 str·s^-1^) (Figure 2, A, B, and C). From V̇O_2max_ (4185.3 ± 686.1 mL min^-1^, 95%CI: 3876.8–4493.8 mL min^-1^) and vV̇O_2max_ (1.42 ± 0.04 m s^-1^), V̇O_2_ and velocity at GET and RCP obtained 70.6% ± 9.4% (95%CI: 66.3%–74.8%) and 82.4% ± 9.2% (95%CI: 78.3%–86.6%) of V̇O_2max_ and 84.6% ± 3.6% (95%CI: 83.0%–86.2%) and 91.9% ± 3.8% (95%CI: 90.1%–93.6%) of vV̇O_2max_, respectively (Figure 2). The relative values of vCp (116.9 ± 9.7 %vV̇O_2max_, 95%CI: 112.6%–121.3%) and V̇O_2_Cp (129.7 ± 20.2 %V̇O_2max_, 95%CI: 120.7%–138.8%) showed a tendency to demarcate a race pace above V̇O_2max_, whereas vSLslope (86.4 ± 8.9 %vV̇O_2max_, 95%CI: 82.4%–90.4%) and V̇O_2_SL_slope_ (75.3 ± 15.4 %V̇O_2max_, 95%CI: 68.4%–82.2%) are demarcating a race pace between GET and RCP limits, and vSR_slope_ (77.9 ± 12.1%vV̇O_2max_, 95%CI: 72.4%–83.3%) and V̇O_2_SR_slope_ (61.7 ± 17.0%V̇O_2max_, 95%CI: 54.0%–69.3%) demarcated the swimming intensity below GET (Figure 2, A, B, and C).

The swimming cost calculated at vSL_slope_ (0.89 ± 0.19 kJ∙m^-1^, 95%CI: 0.80–0.97), vSR_slope_ (0.80 ± 0.18 kJ∙m^-1^, 95%CI: 0.72–0.88), and vCp (1.13 ± 0.20 kJ∙m^-1^, 95%CI: 1.04–1.22) evidenced significant correlations with GET (r = 0.73 [strong], 95%CI: 0.41–0.89, SP = 0.97; r = 0.57 [moderate], 95%CI: 0.16–0.82, SP = 0.76; and r = 0.60 [moderate], 95%CI: 0.20–0.83, SP = 0.80, respectively). Also, the swimming cost at vSLslope and vCp correlated with both RCP (r = 0.63 [moderate], 95%CI: 0.24–0.84, SP = 0.85; and r = 0.69 [moderate], 95%CI: 0.34–0.87, SP = 0.94, respectively) and VO_2max_ (r = 0.70 [strong], 95%CI: 0.37–0.88, SP = 0.94; and r = 0.79 [strong], 95%CI: 0.53–0.92, SP = 0.99, respectively). Correlations to the velocity during the 400 m performance were observed for vCp, vGET, and vRCP (r = - 0.59 [moderate], 95%CI: −0.82—−0.19, SP = 0.80; r = 0.61 [moderate], 95%CI: 0.22–0.83, SP = 0.83; and r = 0.60 [moderate], 95%CI: 0.20–0.82, SP = 0.81, respectively).

Table 1 shows the percentages of GET, RCP, Cp, SL_slope_, and SR_slope_ relative to vV̇O_2max_ and V̇O_2max_. The percentage where SR tends to increase (SR_slope_) differs from the percentage where GET, RCP, Cp, and SL_slope_ are located relative to vV̇O_2max_ and V̇O_2max_.

**TABLE 1:** Aerobic variables and swimming stroke profiles during the incremental step-test.

	Mean	SD
V̇O_2max_ (ml kg^-1^ min^-1^)	63.3	7.7
vV̇O_2max_ (m s^-1^)	1.41	0.04
GET (%V̇O_2max_)	70.6^a^ ^♦^	9.4
vGET (%vV̇O_2max_)	84.6^b^ ^⋄^	3.6
RCP (%V̇O_2max_)	82.4^a^ ^♦^	9.2
vRCP (%vV̇O_2max_)	91.9^⋄^	3.8
Cp (%V̇O_2max_)^♦^	129.7^a^ ^●^	20.2
vCp (%vV̇O_2max_)^⋄^	116.9^b^ ^,^ ^c^	9.7
SL_slope_ (%V̇O_2max_)^●^	75.3^a^ ^♦^	15.4
vSL_slope_ (%vV̇O_2max_)^c^	86.4^b^ ^⋄^	8.9
SR_slope_ (%V̇O_2max_)^a^	61.7^♦^	17.0
vSR_slope_ (%vV̇O_2max_)^b^	77.9^⋄^	12.1

V̇O_2max_: maximal oxygen uptake; vV̇O_2max_: velocity at V̇O_2max_; GET: gas exchange threshold; vGET: velocity at GET; RCP: respiratory compensation point; vRCP: velocity at RCP; Cp: crossing point; vCp: velocity at Cp; SL_slope_: slope of SL; vSL_slope_: velocity at the slope of SL; SR_slope_: slope of SR; vSR_slope_: velocity at the slope of SR.

♦Different from Cp (%V̇O_2max_) with significance at *p* < 0.05.

^⋄^Different from vCp (%vV̇O_2max_) with significance at *p* < 0.05.

^●^Different from SL_slope_ (%V̇O_2max_) with significance at *p* < 0.05.

^a^ Different from SR_slope_ (%V̇O_2max_) with significance at *p* < 0.05.

^b^ Different from vSR_slope_ (%vV̇O_2max_) with significance at *p* < 0.05.

^c^ Different from vSL_slope_ (%vV̇O_2max_) with significance at *p* < 0.05.

No differences were observed where SL tends to decrease (SL_slope_) with %vV̇O_2max_ and %V̇O_2_ at GET, and %V̇O_2_ at RCP, but SL_slope_ differed from %vV̇O_2max_ and %V̇O_2_ at Cp. The results show that Cp localization at %vV̇O_2max_ and %V̇O_2max_ differed from all other physiological (GET and RCP) and stroke (SR_slope_, SL_slope_) references in the maximal incremental step-test.

## 4 Discussion

The current study investigated the association between SL and SR profiles with V̇O_2_ responses during an incremental test performed until exhaustion in front crawl swimming. The highlighted findings are as follows: i) SL and SR slopes did not demarcate similar %V̇O_2max_ or %vV̇O_2max_ responses; ii) while SR increases over moderate-to-heavy-exercise domains (i.e., through GET), the SL response tended to slightly decrease or remain unchanged at peak values; iii) the SL started to drop in the heavy-exercise domain (i.e., between GET and RPC), in which the slope is more costly than SR_slope_ and is strongly correlated to GET, RCP, and V̇O_2max_; and iv) Cp (i.e., the projected point where the reduction in SL and the increase in SR crossed) suggests a swimming condition corresponding to supramaximal exercise intensities (e.g., above V̇O_2max_) and also correlated to GET, RCP, and V̇O_2max_. The observed second-order polynomial (quadratic function) relationships between SL and SR with the swimming velocity and V̇O_2_ response, as well as between cost and swimming velocity, are consistent with previous results (Seifert et al., 2007; Barbosa et al., 2008; Carvalho et al., 2020).

The increase of SR without impairing SL aligns with the statement that SR can increase proportionally to the velocity and energy demand, adjusting stroke mechanics, while maintaining an uncostly profile (Barbosa et al., 2008; Massini et al., 2021). Indeed, the current study observed that this increase in SR tends to occur before reaching the velocity or V̇O_2_ corresponding to GET despite the positive correlation between SR and GET. These findings partially support previous studies reporting similar locations and bivariate associations (Carvalho et al., 2020). Moreover, values previously reported as references for SL (2.46 ± 0.42 m∙str^−1^) and SR (0.48 ± 0.09 str∙s^-1^) slopes in competitive swimmers at a standard power output of 1000 W (±2.861 lO_2_∙min^-1^) (Toussaint and Hollander, 1994) closely resemble the values observed in the current study for SL_slope_, SR_slope_, and GET, although they are not aligned with each other, as suggested. This result strengthens the need to seek individual references for training planning and control since the misalignment between SL_slope_, SR_slope_, and GET suggests that a particular training zone for improving swimming economy exists between SR_slope_ and SL_slope_, which falls somewhat below and above GET. This range is consistent with the exercise zone to improve endurance, i.e., z1–z2, according to González-Ravé et al. (2021).

In turn, when SL starts to drop, there is a critical demand for energetic releasing, which increases the swimming cost. Therefore, SL_slope_ determines a costly adjustment in stroke mechanics that is interesting to be delayed since swimming intensity is reaching the boundary between the heavy- and severe-exercise domain (i.e., RCP, according to Pessôa Filho et al., 2012). This finding aligns with the reports that an increase in anaerobic contributions occurring as swimming intensity reaches heavy and severe exercise intensities (de Jesus et al., 2016; Carvalho et al., 2020) and an increased hydrodynamic drag (Seifert et al., 2014) are both factors contributing to SL and SR crossing trend. Moreover, the correlation of the swimming cost at SL_slope_ with GET, RCP, and V̇O_2max_ supports the statement delaying SL reduction can ensure and increase in SR (and, thus, swimming velocity) within exercise zones, where aerobic energy supply predominates (e.g., bellow GET) (Barbosa et al., 2008), In addition, delaying the drop in SL can improve tolerance during middle-distance performance (Alberty et al., 2009), as well as positively affect aerobic endurance and power (Wakayoshi et al., 1993). Hence, the current findings regarding V̇O_2_ responses at SL_slope_ suggest that it demarcates an ideal exercise zone for the enhancement of both anaerobic threshold and aerobic power, which are physiological indexes that influence performance during 400, 800, and 1500 m races (Toussaint and Hollander, 1994).

The crossing point between SL and SR profiles was estimated as a mathematical projection in the current study. However, the cost at the Cp showed a similar correlation level with the physiological indexes of an aerobic incremental exercise (i.e., GET, RCP, and V̇O_2max_) as observed with the cost at SL_slope_. Although the meaning of these correlations differed from that discussed for SL_slope_, no previous reports support the meaning of Cp on the physiological response. Therefore, the current study observed that Cp relies on supramaximal exercise intensity (i.e., above 100%V̇O_2max_), more precisely at ∼130% and ∼117% of the V̇O_2max_ and vV̇O_2max_, respectively, which correspond to exercise zones that are relevant to the 100 m race, and with high anaerobic (phosphagens and glycolytic) requirements for both sexes (Massini et al., 2021). Moreover, Cp was the only variable of stroke mechanics showing a correlation to v400 m.

Indeed, Cp can be interpreted as the point at which SL and SR show the same values; thus, Cp is probably coincident with the point where the propelling phases of both arms are superimposed. In addition, this point of arm coordination can be observed either during sprint performance in 100 m (Seifert et al., 2007) or in overcoming mechanical power declining due to exhaustion during the 400 m race (Alberty et al., 2009). Therefore, the current study speculates that the increase of Cp might be associated with the ability to increase SR, while SL decreases slowly, positively affecting both velocity development and tolerance during short- and middle-distance races, respectively. Moreover, the correlation of the cost at Cp with GET, RCP, and V̇O_2max_ also indicates that this ability is present among swimmers with high aerobic contributions during submaximal and maximal swimming intensities. In other words, it suggests reduced anaerobic glycolysis activation and delayed acid–base disturbance at faster submaximal and maximal velocities (Troup, 1986; Toussaint Hollander, 1994; Wakayoshi et al., 1995; Pessôa-Filho et al., 2017; Almeida et al., 2020).

The current study presents some limitations concerning the control of the maturational stage of the swimmers since body growth and different anthropometric dimensions can influence stroke mechanics (Alves et al., 2022), as different height and arm spam seem to be positively correlated with SL but not with SR (Tijani et al., 2019). Another limitation is the swimming cost estimate without considering the contribution of anaerobic sources during swimming intensities above RCP, which should increase the cost (Pessôa Filho et al., 2017), and, therefore, be considered in future studies. Additionally, the role of blood lactate levels could be addressed in future studies, as it might provide physiological information about the meaning of Cp.

## 5 Conclusion

The findings suggest that SL_slope_ and SR_slope_ showed independent profiles from each other when compared with the V̇O_2_ response and velocity increments. In addition, the swimming cost at SL_slope_ correlated to GET, RCP, and V̇O_2max_, while SR_slope_ correlated only with GET. This association suggests that the range of swimming velocities from SR_slope_ to SL_slope_ (i.e., slightly below or just above GET) forms an economical zone (i.e., moderate aerobic pace recommended to improve long-term endurance ability). On the other hand, SL_slope_ might be considered the metabolic reference aforementioned in which the aerobic pace demands a costly stroke profile. Therefore, swimming at such heavy to severe intensities (i.e., close to or above RCP) should be suitable to enhance the anaerobic threshold and aerobic power. Finally, considering that Cp is located at supramaximal swimming intensity (i.e., higher than the velocity or V̇O_2_ at maximal aerobic power), it probably relies on the range of exercise intensities with limited aerobic contribution, hence suggesting a swimming pace with a high demand upon anaerobic energy sources. However, improvements in Cp might be associated with enhanced aerobic energy contribution during submaximal and maximal swimming intensities.

## Data Availability

The raw data supporting the conclusion of this article will be made available by the authors, without undue reservation.
